# NK1 antagonists attenuate tau phosphorylation after blast and repeated concussive injury

**DOI:** 10.1038/s41598-021-88237-0

**Published:** 2021-04-23

**Authors:** Frances Corrigan, Ibolja Cernak, Kelly McAteer, Sarah C. Hellewell, Jeffrey V. Rosenfeld, Renée J. Turner, Robert Vink

**Affiliations:** 1 Clinical and Health Sciences, University of South Australia, GPO Box 2471, Adelaide, SA 5001 Australia; 2 Discipline of Anatomy and Pathology, Adelaide Medical School, University of Adelaide, Adelaide, Australia; 3 Department of Biomedical Sciences, Mercer University School of Medicine, Macon, GA USA; 4 Department of Surgery, Monash University, Melbourne, Australia; 5 Department of Neurosurgery, The Alfred Hospital, Melbourne, Australia

**Keywords:** Diseases of the nervous system, Preclinical research

## Abstract

Exposure to repeated concussive traumatic brain injury (TBI) and to blast-induced TBI has been associated with the potential development of the neurodegenerative condition known as chronic traumatic encephalopathy (CTE). CTE is characterized by the accumulation of hyperphosphorylated tau protein, with the resultant tau tangles thought to initiate the cognitive and behavioral manifestations that appear as the condition progresses. However, the mechanisms linking concussive and blast TBI with tau hyperphosphorylation are unknown. Here we show that single moderate TBI, repeated concussive TBI and blast-induced mild TBI all result in hyperphosphorylation of tau via a substance P mediated mechanism. Post-injury administration of a substance P, NK1 receptor antagonist attenuated the injury-induced phosphorylation of tau by modulating the activity of several key kinases including Akt, ERK1/2 and JNK, and was associated with improvement in neurological outcome. We also demonstrate that inhibition of the TRPV1 mechanoreceptor, which is linked to substance P release, attenuated injury-associated tau hyperphosphorylation, but only when it was administered prior to injury. Our results demonstrate that TBI-mediated stimulation of brain mechanoreceptors is associated with substance P release and consequent tau hyperphosphorylation, with administration of an NK1 receptor antagonist attenuating tau phosphorylation and associated neurological deficits. NK1 antagonists may thus represent a pharmacological approach to attenuate the potential development of CTE following concussive and blast TBI.

## Introduction

The accumulation of perivascular, hyperphosphorylated tau at the base of sulci following repeated concussive TBI is a hallmark of CTE, with its initial anatomical distribution thought to be associated with local mechanical damage^1^. Mechanosensation in the brain is mediated, in part, by members of the transient receptor potential (TRP) family of channels^2,3^, namely TRPV1, TRPV4 and TRPA1, whose activation is linked to the release of the neuropeptide substance P^2,4^. Substance P preferentially binds to the tachykinin NK1 receptor^5^ and has been shown to increase kinase activity^6–8^, including that of JNK, Akt and ERK1/2. Notably these kinases, amongst others, have been associated with tau phosphorylation^9^. We therefore hypothesized that TBI-induced mechanical activation of brain TRP receptors might lead to increased substance P release, subsequent activation of neuronal NK1 receptors and ultimately hyperphosphorylation of tau. We have used experimental models of both concussive and blast injury to investigate this hypothesis.

## Results

**Figure 1:**
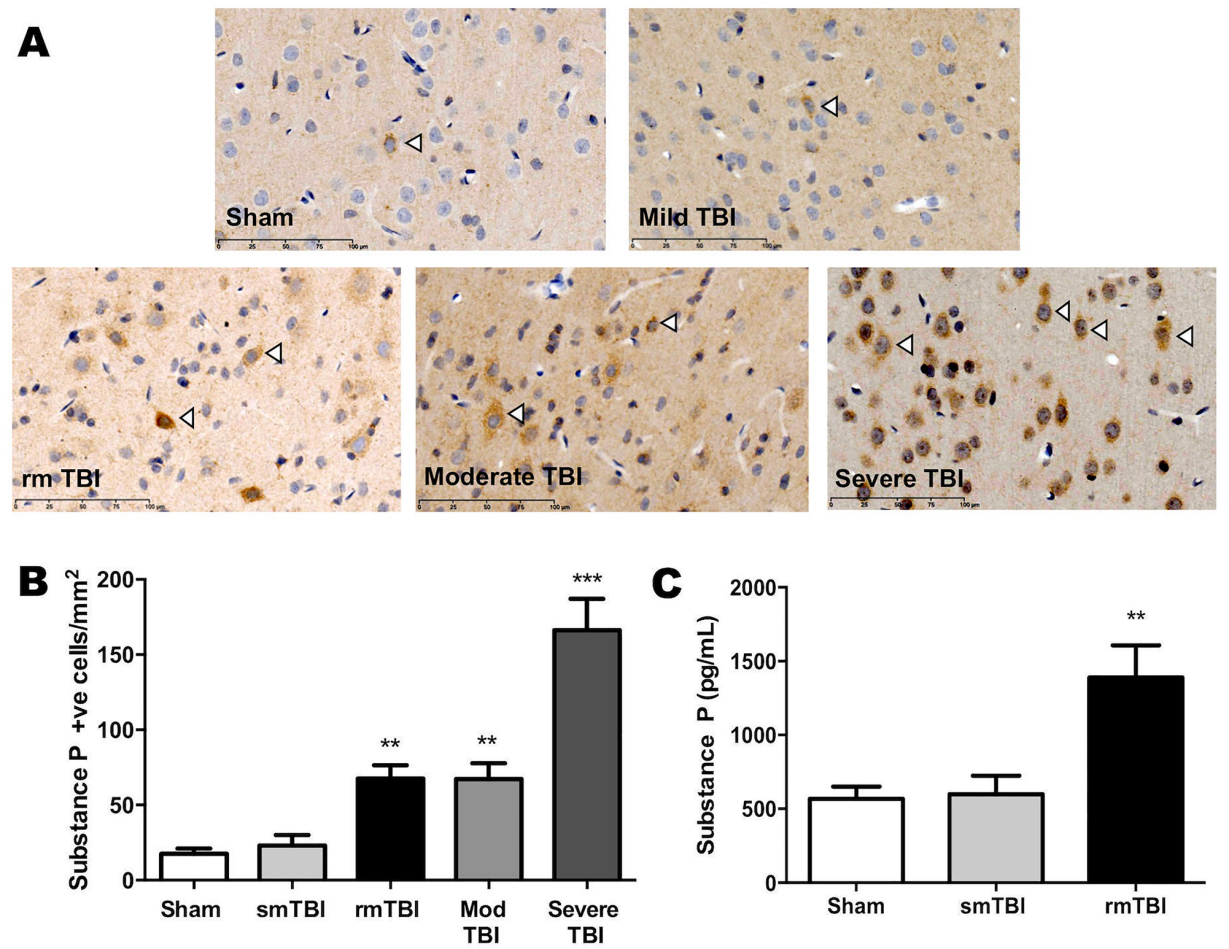
Increases in substance P immunoreactivity following diffuse TBI in rats. Immunohistochemistry **(A)** typically demonstrates the presence of substance P positive cortical neurons (arrowed) immediately below the impact site at 24 h after the final injury. While no significant increase was observed after a single mild TBI (smTBI), clear increases in substance P immunoreactivity were observed with increased intensity of the injury and with repeated exposure to the mild injury (rmTBI). Cell counts **(B)** and ELISA assay **(C)** confirm the immunohistochemistry findings. Mean ± SEM; n = 5–8/group; ** = p < 0.01, *** = p < 0.001 versus sham and smTBI.

Following a single, moderate to severe diffuse TBI in rats^10^, there was an injury dependent increase in substance P immunoreactivity in the cortex immediately below the TBI impact site at 24 h after injury (Fig. 1A). Such increases in cortical substance P immunoreactivity have been previously reported by us following moderate to severe TBI in both animal and humans, and have been identified as being predominantly perivascular in origin^4,10^. In the present study, no significant increase in substance P occurred after a single mild (concussive) TBI (Fig. 1A), although there was a significant increase (p < 0.01) following repeated mild TBI in rats delivered as 3 injuries each at 5 days apart^11^, such that cortical substance P immunoreactivity after the 3^rd^ TBI was similar to that following a single moderate injury (Fig. 1B). Cortical ELISA assay confirmed the immunohistochemistry findings following repeated mild TBI (Fig. 1C). These observations are consistent with previous reports demonstrating that substance P release is amplified with increasing intensity of stimulus or with repeated stimulation^5^, allowing substance P to diffuse away from the site of release and activate up to 5 times more neurons expressing NK1 receptors. This is particularly relevant to perivascular neurons where substance P would radiate away from the vasculature to activate surrounding neurons expressing NK1 receptors.

**Figure 2:**
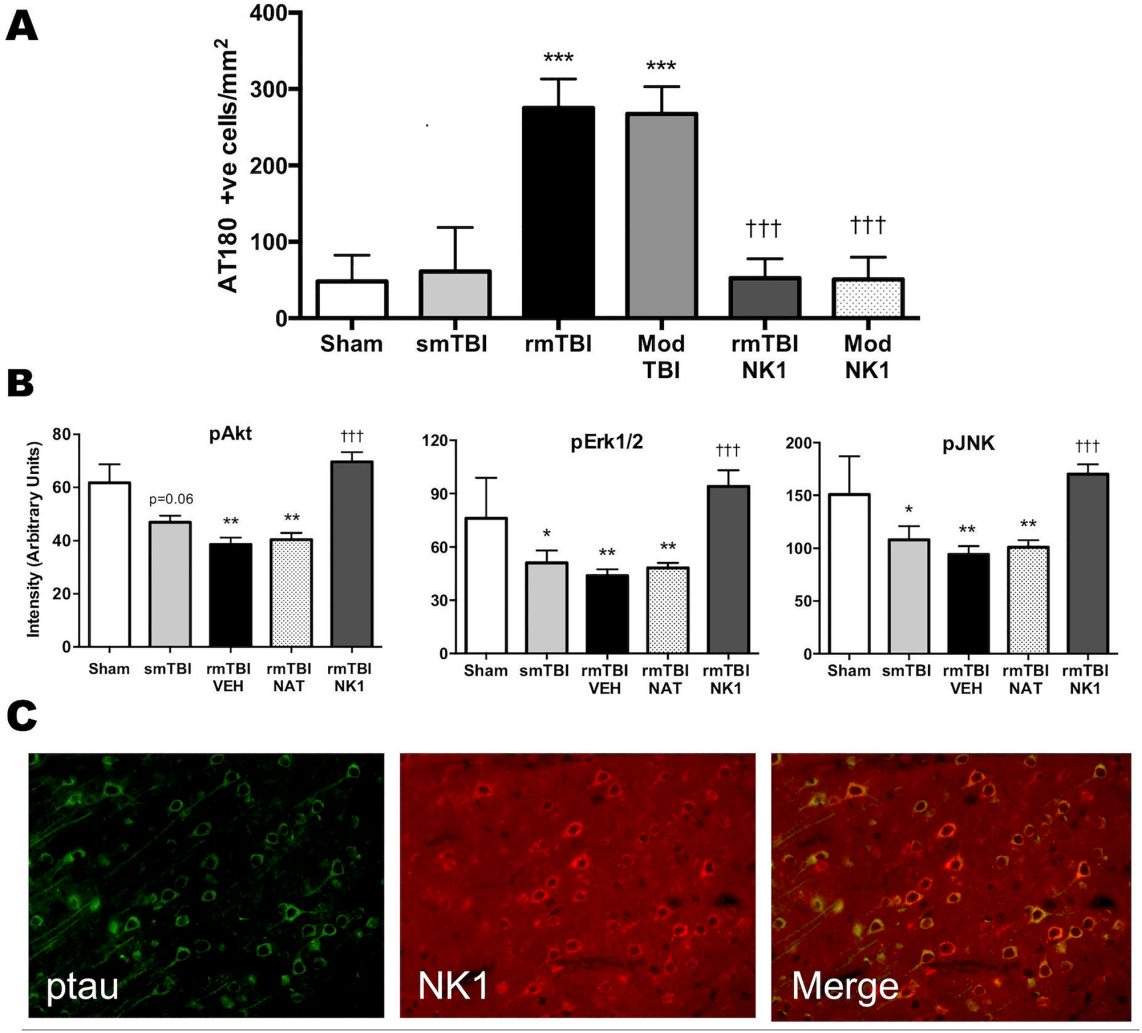
**(A)** Changes in cortical tau phosphorylation (AT180) at 24 h following single, mild TBI (smTBI), repeated mild TBI (rmTBI) or moderate TBI with and without NK1 antagonist (EUC-001) treatment. Mean ± SEM; n = 4–6/group; *** = p < 0.001 versus sham and smTBI; ^†††^ = p < 0.001 versus no treatment. **(B)** Changes in phosphorylated kinase levels at 24 h following smTBI and rmTBI, with and without administration of the NK1 antagonist, EUC-001. NAT (n-acetyl-tryptophan) is a non, cell-permeable NK1 antagonist. Note that only the cell permeable NK1 antagonist EUC-001 returned post-injury phosphorylated kinase levels back to sham (non-injured) levels. Mean ± SEM; n = 5–6/group; * = p < 0.05, ** = p < 0.01 versus sham; ^†††^ = p < 0.001 versus vehicle and NAT treated animals. **(C)** Typical confocal microscopy images of cortical neurons expressing phosphorylated tau (AT180), NK1 receptors, and their colocalization in the merged image. Images were obtained at 24 h following a single moderate TBI.

Cortical tau hyperphosphorylation was also evident 24 h after TBI. As with the substance P results, a single mild TBI did not result in significant cortical tau phosphorylation, while repeated mild TBI resulted in a significant increase in tau phosphorylation (p < 0.001), with levels being similar to that observed following a single moderate injury (Fig. 2A). Notably, administration of an NK1 antagonist, EUC-001^12^, significantly attenuated (p < 0.001) the tau phosphorylation following both single moderate and repeated mild TBI. We have previously shown that induction of single and repeated mild TBI induces changes in kinase and phosphatase activity that may be associated with increased phosphorylation of tau protein^13^. Moreover, others have shown that substance P increases kinase activity^6–8^, including that of Akt, JNK and ERK1/2. Consistent with these observations, the NK1 antagonist EUC-001 reversed any changes in the phosphorylation of the Akt, JNK and ERK1/2 kinases at 24 h after repeated mild TBI back to a sham (non-injured) state (Fig. 2B). Notably, total levels of the kinases did not change after TBI (data not shown). Of interest is the observation that a cell-impermeable NK1 antagonist, n-acetyl L-tryptophan (NAT), previously shown by us to be efficacious when administered early after moderate to severe TBI^14^ had no effect on any injury-induced changes in kinase phosphorylation after repeated mild TBI as compared to the cell-permeable EUC-001 (Fig. 2B). This suggests that there is a need for the NK1 antagonist to cross the intact BBB and bind to the neuronal NK1 receptor to be effective in preventing tau phosphorylation. Confocal microscopy images obtained after TBI confirmed that NK1 receptors were indeed co-localized to neurons that demonstrated increased phosphorylated tau after TBI (Fig. 2C).

**Figure 3:**
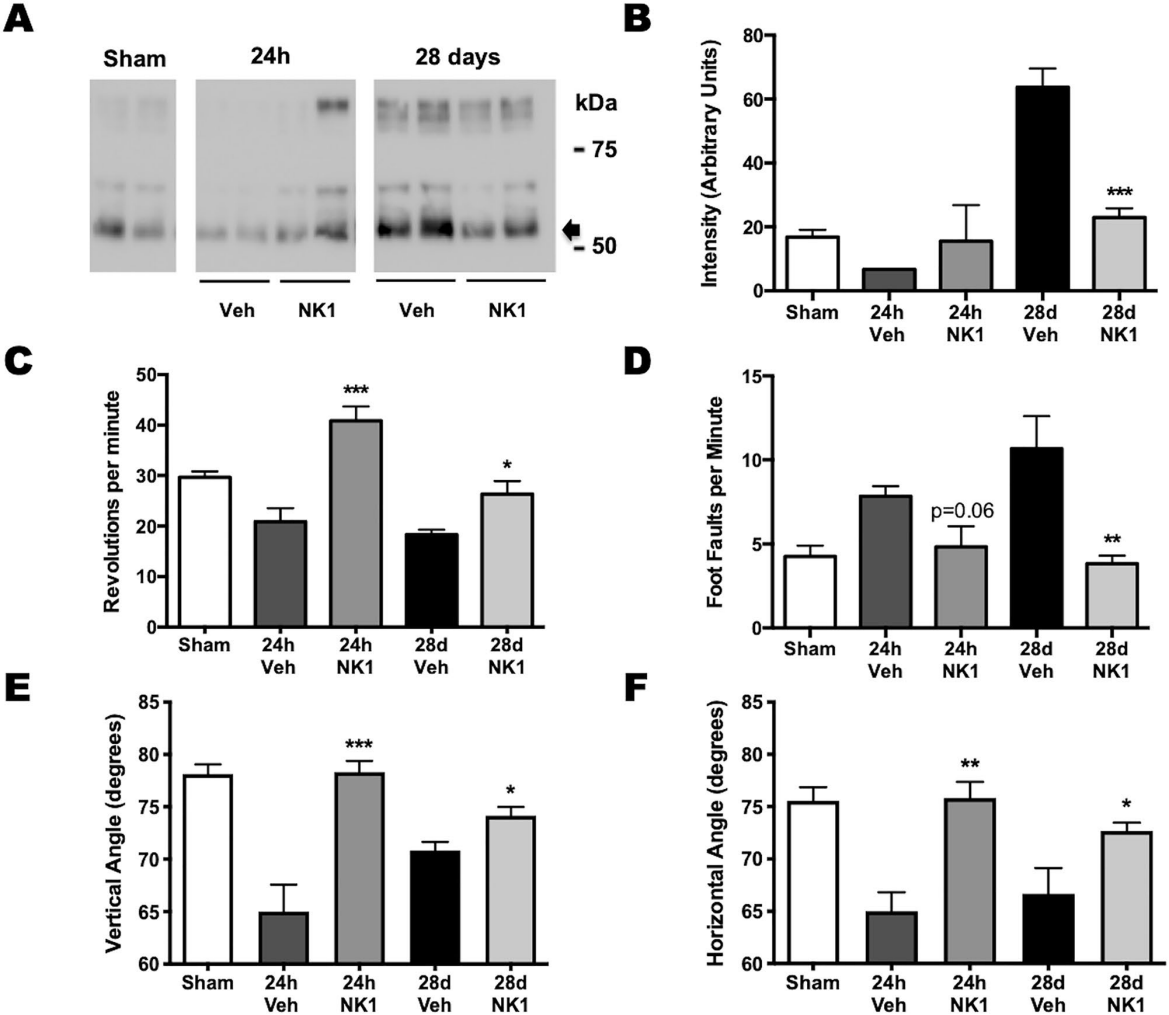
Changes in tau phosphorylation (serine 396, 50–60 kDa; arrow) and neurological outcome at 24 h and 28 days following blast TBI in mice, with and without administration of the NK1 antagonist EUC-001. Western blot images **(A)** and their quantitation **(B)** demonstrate a significant increase (p < 0.001) after injury versus shams, while treatment with the NK1 antagonist reduced the tau phosphorylation back to sham levels. Mean ± SEM; n = 6/group; *** = p < 0.001 versus no treatment. Improvements in neurological outcome were noted in rotarod performance **(C)**, foot faults on the beam walk **(D)**, and on the angleboard in the vertical **(E)** and horizontal position **(F)**. Mean ± SEM; n = 6/group; * = p < 0.05, ** = p < 0.01, *** = p < 0.001 versus vehicle treated animals at the same time point.

Tau hyperphosphorylation and development of CTE has also been reported in military personnel after exposure to blast^1^. Accordingly, we examined the efficacy of NK1 antagonists to attenuate tau phosphorylation in an experimental mouse model of mild blast-induced TBI^15^. Following blast, a significant increase (p < 0.001) in brain tau phosphorylation (serine 396 epitope) was indeed observed at 28 days after injury (Fig. 3A), although in the present study, no significant increase was detected at 24 h (see supplementary information for full blots). There were no changes in total tau levels at either time point (data not shown). Administration of the NK1 antagonist at 30 min after blast injury significantly reduced (p < 0.001) levels of tau phosphorylation at 28 days back to sham levels (Fig. 3B). Administration of the NK1 antagonist also significantly improved (0.0001 < p < 0.05) neurological outcome at both 24 h and 28 days on the rotarod, vertical and horizontal angleboard tests, and the 28-day outcome on the beamwalk test (Fig. 3C–F). Although the beamwalk performance at 24 h was improved by NK1 antagonist administration, this was marginally statistically insignificant in the current study (p = 0.06). Notably, the improvements in neurological outcome at 24 h occurred in the absence of any significant change in brain phosphorylated tau, suggesting that neurological outcome at this time point of 24 h was unrelated to the status of tau. In contrast, the improvement in neurological outcome at 28 days was clearly associated with decreased presence of phosphorylated tau.

**Figure 4:**
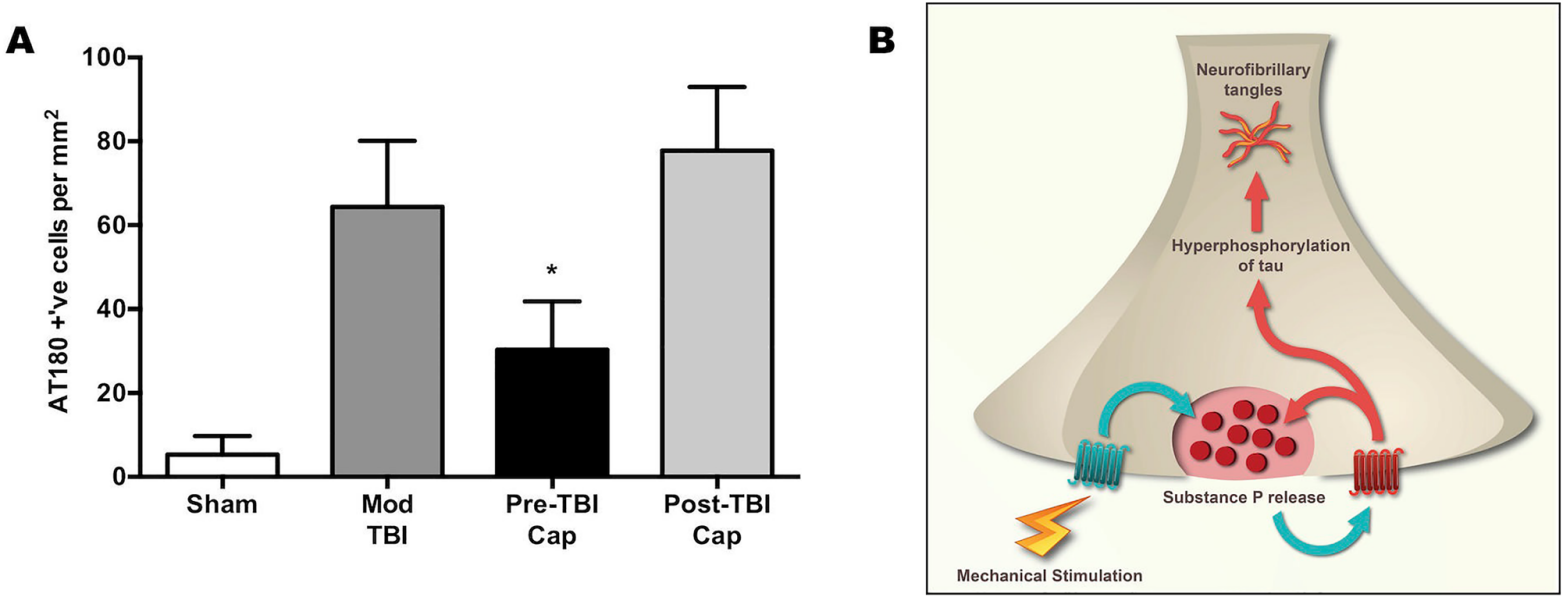
**(A)** Effects of the TRPV1 antagonist, capsazepine (Cap), on 24 h tau phosphorylation (AT180) when administered 30 min prior to or 30 min following moderate TBI. Capsazepine administration significantly reduced tau phosphorylation only when administered prior to injury. Mean ± SEM; n = 6/group; * = p < 0.05 versus nontreated animals. **(B)** Schematic summarizing mechanisms associated with tau phosphorylation following concussive and blast brain injury. Mechanical stimulation of transient receptor potential (TRP) receptors initiates substance P release, which binds to NK1 receptors. NK1 receptor stimulation activates kinases that then phosphorylate tau, leading to neurofibrillary tangles. Repeated or high intensity NK1 receptor activation can also promote further substance P release^5^.

Finally, we examined the role of mechanosensitive TRP channels in tau phosphorylation. While TRPV1, TRPV4 and TRPA1 all promote substance P release and respond to mechanical stimuli^3^, the TRPV1 receptor and its linkage to perivascular substance P release and kinase activation has been the most thoroughly characterized^16^, including the identification of selective pharmacological antagonists. Moreover, the TRPV1 receptor has been shown to activate in response to mechanical stimulation at the brain-fluid vascular interface^2,3^, including to changes in blood pressure^17^ which have been well described after TBI. Despite being just one of three TRP mechanoreceptors linked to neuronal substance P release, administration of the TRPV1 antagonist, capsazepine, 30 min prior to moderate single TBI significantly attenuated (p < 0.05) cortical tau phosphorylation at 24 h after the insult (Fig. 4A). In contrast, administering the TRPV1 antagonist at 30 min after the insult had no effect on tau phosphorylation, suggesting that post-insult inhibition of mechanosensitive TRP channels is of no benefit in halting the initiated injury cascade. Figure 4B summarizes the proposed injury cascade whereby mechanical stimulation of TRP channels leads to tau phosphorylation via substance P release and activation of NK1 receptors. It has been previously shown that repeated or high intensity NK1 receptor activation promotes further substance P release^5^.

## Discussion

We have previously demonstrated that in diffuse, moderate TBI, treatment with an NK1 antagonist reduces acute neuronal cell death and improves associated neurological outcome; these actions are primarily mediated by the antagonist reducing the substance P induced increase in blood brain barrier permeability^10,14^. Blood brain barrier permeability has also been reported to increase following a series of repeated concussive TBIs^18^, which is consistent with our current observation of profoundly increased substance P release after repeated mild TBI. However, we also demonstrate that the non, lipid-soluble NK1 antagonist, NAT, was ineffective in altering kinase profiles after repeated mild TBI as compared to the cell-permeable NK1 antagonist, EUC-001, suggesting that the barrier opening after each individual mild TBI was insufficient to permit central penetration of the non, lipid-soluble antagonist. Similar results have been described comparing the efficacy of lipid-soluble NK1 antagonists and non, lipid-soluble antagonists at later time points after moderate to severe TBI^14^, time points when increased blood brain barrier permeability is still apparent, but to lesser size molecules^14^. To avoid potential inefficacy through lack of central penetration, we now routinely utilize a lipid-soluble NK1 antagonist^12^ in all of our studies of TBI.

We have previously reported acute/sub-acute increases in brain phosphorylated tau following both a single moderate to severe injury and repeated mild injury^11,13^ as well as following blast injury^15^ and have replicated those results here. The acute/sub-acute increase in hyperphosphorylated tau contributes to secondary injury after TBI by promoting prolonged detachment of tau from microtubules and enhancing its aggregation. The normal function of tau is to promote stabilization of microtubules with repeated rounds of phosphorylation/ dephosphorylation to allow detachment and movement of cargo. When tau becomes hyperphosphorylated it remains detached, destabilizing microtubules and impairing axonal transport which is vital for vesicles and organelles to reach the synapse^19^. This leads to deterioration of synapses, with eventual retrograde degeneration. Such ongoing tau-induced degeneration is consistent with reports of ongoing neuronal loss following single diffuse TBI^20,21^, repeated mild TBI^22^ and following blast TBI^15^. Detached hyperphosphorylated tau is also more likely to aggregate, first as oligomers and then as paired helical filaments and ultimately neurofibrillary tangles. The early tau oligomers are thought to be particularly toxic, acting as templates for the misfolding of native tau, seeding the spread of toxic tau species^23^, as well as directly disrupting synaptic function^24^. Preventing acute alterations in tau phosphorylation may thus have long-term benefits after TBI. Indeed, complete ablation or partial reduction of tau prevented deficits in spatial memory on the Barnes Maze at one month after repeated TBI^25^.

Given the detrimental effects of increased hyperphosphorylated tau following TBI, it is important to identify treatments that target this secondary injury factor. Here we show that preventing substance P signaling, either by blocking its NK1 receptor or by preventing its release using a TRPV1 mechanoreceptor antagonist, reduces levels of tau phosphorylation after TBI. This was associated with a restoration of kinase levels (Akt, ERK1/2, JNK) to normal (sham) levels. While repeated injury in the current study was associated with a decrease in the phosphorylated active forms of these kinases at the 24 h timepoint, kinase activation is transient^26^ and the acute increases immediately after TBI may have been missed at the 24 h time-point, resulting in the rebound decrease noted here after the phosphate group has been donated to the tau protein. Apart from direct modulation of kinases, prevention of other secondary injury factors, including axonal injury and inflammation, via blockade of substance P^4^ could also be important in reducing tau phosphorylation after TBI. The mechanical stress associated with TBI is known to alter the behavior of tau proteins, causing them to act more stiffly than usual, leading to mechanical breakdown of microtubules and release of tau^27^. Axonal injury continues beyond this initial mechanical insult, with mechanoporation of the axonal membrane allowing influx of calcium and activation of calpain pathways that degrade the cytoskeleton including microtubules^28^, permitting further release of tau and its subsequent phosphorylation. TBI also leads to upregulation of an acute inflammatory response^4^, including pro-inflammatory cytokines like interleukin-1 and interleukin-6, which have been shown to promote phosphorylation of tau^29^. Blockade of substance P via administration of an NK1 antagonist following moderate to severe TBI has previously been shown to reduce axonal injury^14^. Furthermore, substance P is known to amplify the classical inflammatory response, promoting microglia and astrocyte activation and the release of pro-inflammatory mediators including the cytokines interleukin-1 and interleukin-6^4,8^. Thus, this inflammatory response may have been attenuated following administration of an NK1 antagonist or capsazepine, to further assist in reduction in tau phosphorylation post-injury.

In this study we have demonstrated that inhibition of substance P signaling prevents the acute increase in tau phosphorylation noted following both single moderate and repeated mild TBI, and the delayed increase seen after single mild blast injury. Alteration in tau phosphorylation is an important secondary injury factor after TBI with hyperphosphorylation of tau promoting its aggregation into oligomers, which are thought to act as seeds for further tau pathology and ongoing neuronal cell injury. While it is yet to be confirmed in larger, gyrencephalic animals that such tau hyperphosphorylation will be concentrated at the base of sulci where the greatest mechanical loading occurs in response to TBI^30^, our present results have nonetheless demonstrated that treatment with an NK1 antagonist acutely after single moderate to severe TBI, repeated mild TBI or mild blast injury may have long-term benefits, including attenuating the potential development of tau pathologies such as CTE.

## Methods

All animal studies were carried out in compliance with the ARRIVE guidelines (http://www.nc3rs.org.uk/page.asp?id=1357) as detailed below.

### Ethical approval

Rat TBI studies were conducted in accordance with guidelines established by the National Health and Medical Research Committee of Australia and undertaken at the University of Adelaide following approval by the institutional Animal Ethics Committee. Mouse blast injury experiments were conducted in accordance with Canadian Council on Animal Care (CCAC) guidelines and were undertaken in the Blast Research Facility of the Combat Casualty Care Division at the Defence Research Department of Canada (DRDC), Suffield following approval by both the University of Alberta and the DRDC Suffield Animal Ethics Committees.

### Rat model of TBI

Male, Sprague–Dawley rats (350-400 g) were housed in a temperature-controlled environment under a 12 h light/dark cycle with uninterrupted access to food and water before being randomly allocated to receive either sham procedures, a single moderate to severe TBI (TBI) or repeated mild TBI under anesthesia, as has been described in detail previously^11^. Animals were anaesthetized via inhalation of 5% isoflurane (Henry-Schein) using a normoxic mixture of air (70% nitrogen, 30% oxygen; BOC), at a rate of one liter per minute. Once absence of reflexes was apparent, animals were maintained on 2% inhalational isoflurane via a nose cone for the duration of the procedure. In brief, for the single moderate injury, a 400 g weight is dropped from 2 m onto a steel disc, whilst for repeated mild TBI, the weight is dropped from 1 m onto the steel disc on days 0, 5 and 10. Induction of three injuries with a 5-day time interval has been previously described as being optimal to produce cumulative long-term functional deficits with repeated mild TBI^11^. Sham animals were anesthetized and underwent surgery without impact, with the sham group containing a mix of single surgery shams and repeated surgery shams (undertaken on days 0, 5 and 10). A 10 cm thick foam cushion decelerates the head after impact, thus producing the acceleration/deceleration injury. After injury, the skin overlying the injury site is sutured and the rats are returned to their home cage. Temperature is maintained throughout all procedures using a water-heated thermostatically controlled heating pad. Sham control animals undergo surgery, but do not receive an impact.

### Mouse model of mild blast injury

Adult male C57Bl/6 mice (~ 10 weeks of age; Charles River Laboratories, Montreal, Quebec, Canada) were housed in temperature-controlled rooms on a 12 h light/dark cycle with access to food and water ad libitum. After a 1-week acclimation period, animals were randomly allocated to receive either sham procedures or mild blast injury as previously described in detail elsewhere^31^. Briefly, a custom-built Advanced Blast Simulator (ABS; 30.5 cm in diameter and 5.79 m in length) located at DRDC Suffield was used to produce a highly reproducible single pulse shock waves tailored to replicate those of explosive blast. Mice were anaesthetized with 3% isoflurane evaporated in 30% oxygen & 70% nitrous oxide in an induction chamber for two minutes, followed by an additional four minutes of administration via nose cone while the animal was secured to the blast holder. When deeply anesthetized (confirmed by the absence of tail, corneal and toe pinch reflexes), the blast holder was dispatched to the ABS and rotated so the mouse was in a bipedal position facing front-on to the blast wave, and the holder advanced into the shock tube a distance of 30 cm (2.4 m from the membrane) so as to expose the animal to a low intensity (10 psi) total blast pressure, approximating mTBI. This exposure was selected because it was the lowest exposure at which we were able to detect sensorimotor dysfunction and pathology of the Purkinje cell layer of the cerebellum, which is a hallmark of clinical and preclinical blast exposure^31^. The end-wave eliminator was then quickly secured and the blast sequence initiated. Sham mice were anaesthetized and secured to the blast holder as for the blast group, without transport to the shock tube. After blast exposure or sham procedure, mice were quickly removed from the holder by cutting the cable ties on one side and placed into a clear Perspex cage for acute neurological monitoring.

The blast-induced TBI model used in this work was developed based on our previously developed and standardized models^15,31^, and the presence of the hallmarks of blast injury such as ecchymosis in the lungs, among others, have been verified by the Yelverton blast scoring system at 24-h time-point^31,32^. This system provides an excellent profile of whole-body damage by combining scores for the extent (E) of organ damage, the grade (G) of organ damage, the severity (ST) of pathology, and the severity depth (SD) to indicate the depth of disruption of the worst pathology observed. These scores are combined in the following formula to reach a final injury severity (IS) score: IS = (E + G + ST) x SD. Examination of whole-body pathology revealed evidence of lung injury consisting primarily of scattered petechial hemorrhages most prominent at the lung periphery. In some cases, small areas of hemorrhage involving less than 10% of the lungs were observed. Similar to the model used in Cernak et al.^31^ and Huber et al. studies^15^, overall, the lung injury fell within the slight score based on the Yelverton blast injury scoring system. Pathological sequelae (manly petechiae and ecchymosis) in the liver and kidneys were also within the slight Yelverton score. As this model reproduced mild BINT, it was not surprising that these structural changes were not observed at the 28-day timepoint.

### Drug treatments

After injury, randomly selected animals in each injury group were administered with either 1 mg/kg of the lipid soluble NK1 antagonist EUC-001^12^ (Eustralis Pharmaceuticals, Melbourne, Australia), 2.5 mg/kg of the non-lipid soluble NK1 antagonist n-acetyl L-tryptophan (Sigma-Aldrich, Castle Hill, Australia), or equal volume saline vehicle. Drugs were administered as a single bolus i.v. in rats and i.p. in mice at 30 min after single injury, or 30 min after each injury induction following repeated mild TBI.

### Group allocation

For examination of the effects of injury on SP expression, animals were allocated to sham, single mild, repeated mild and single moderate to severe injury with perfuse fixation at 24 h post-injury. A separate cohort of sham, smTBI and rmTBI animals were saline perfused and the brain frozen for cortical SP assessment. For analysis of the effects of NK1 antagonism on tau phosphorylation in the impact-acceleration model, animals were allocated to either sham, smTBI, rmTBI, modTBI, rmTBI + NK1 treatment and modTBI + NK1 treatment and perfusion fixed at 24 h post-injury. A separate cohort were allocated to sham, smTBI, rmTBI, rmTBI + NAT or rmTBI + EUC-001, saline perfused and brains fresh frozen for analysis of kinase activation post-TBI. Mouse blast experiments followed a similar group allocation procedure, with mice randomly allocated to sham, single mild blast, or single mild blast + EUC-001. For all assessments, including neurological outcomes and pathology, assessors were routinely blinded to the group allocation.

### Mouse neurological monitoring

At days 1 and 28 after injury, mice were assessed for neurological motor outcome using the inclined plane, rotarod and tapered beam walk tests (n = 6/group). In the angle board test, mice were assessed on their ability to grip onto an inclined board which was covered with a grooved rubber mat. The angle was increased in 5-degree increments, with ability to grip assessed facing vertically (up and down the incline) or horizontally (across the incline). The maximum angle at which a mouse was able to maintain its position for at least 5 s was taken as the score for that animal. In the rotarod test, animals were assessed on their ability to walk on a rotating assembly of rods, where the device speed increases by 3 revolutions per minute every 5 s. Each mouse was given three trials, with the average taken as the final score for revolutions per minute achieved. Animals were pretrained on the device for 1 week prior to induction of injury. For the tapered beam test, mice were required to traverse a one-metre-long beam placed on a 30° incline. At its widest, the beam is 3.5 cm and decreases to 0.5 cm at the tip, with a lowered ledge on both sides to support foot faults. Mice with hind limb gait deficits are unable to keep their balance on the upper portion of the beam, requiring extra support from the ledged portion of the beam. Mice were given one training session and one trial per testing period, with the number of hind limb foot slips to the lower portion recorded.

### Biochemical analysis

Rats were terminally with isoflurane prior to transcardial perfusion with saline. The brains were removed, the cortex under the impact site dissected and snap frozen. Protein was then extracted, with protein concentration estimated with a Pierce BCA Protein Assay (Thermo Scientific, Waltham, MA, USA). Levels of SP within homogenates were measured via a SP Parameter Assay Kit (KGE007, R&D Systems, Minneapolis MN, USA) as per the manufacturer’s protocol, with all samples measured in triplicate (n = 5–6/group). Analysis of kinases (Akt, ERK1/2 and JNK) was performed using custom made Bio-Plex Pro Cell Signaling Assay kits (Bio-Rad, Hercules, CA, USA) as per the manufacturer’s protocol (typically n = 5/group). The plate was then loaded into a Bio-Plex MAGPIX reader (Bio-Rad) and the output read by Bio-Plex Manager Software (Bio-Rad). Readings were then collated and analyzed statistically.

### Western blotting

Brains of blast-exposed and sham control mice were assessed for tau and its phosphorylation by Western blot as previously described in detail elsewhere^15^. Briefly, protein extracts were prepared from dissected brains (n = 6/group) consisting predominantly of cortex but also including underlying subcortical thalamic regions. TGX gels (4–20%; Bio-Rad) were then loaded with 20 μg/lane and probed after overnight incubation (4 °C) with antibodies for phosphorylated tau AT8 (Thermoscientific) and serine 396 (Life Technologies, Grand Island NY, USA). Probes for total tau (tau5) and pyruvate kinase (Rockland, Gilbertsville, PA) were conducted after stripping. Target bands were densitometrically analyzed with ImageQuant TL (GE, Piscataway, NJ), with tau epitope levels standardized to total tau level and expressed relative to sham.

### Immunohistochemistry

Rats (typically 6/group) were terminally anaesthetised and transcardially perfused with 10% formalin before their brains were removed and stored in 10% formalin until use. Five μm sections were collected at 250 μm intervals, representing the region from Bregma − 2.5 to 3 mm. Sections were then stained for levels of tau phosphorylation (AT180, 1:1000, MN1040, Thermo Fisher) or SP (1:1000, ab10353, Abcam, Cambridge MA, USA). Following dewaxing, endogenous peroxidases were blocked with methanol/hydrogen peroxide (0.5%), followed by antigen retrieval in citrate buffer. Sections were then incubated with 30% normal horse serum for 1 h, prior to incubation overnight at room temperature with the specific primary antibody. The next day, the appropriate biotinylated secondary antibody (1:250, Vector Laboratories, Burlingame CA, USA) was applied for 30 min, followed by streptavidin horseradish peroxidase for 60 min, with the bound antibody detected with 3,3-diaminobenzidine tetrahydrochloride (Sigma-Aldrich). Sections were counterstained with hematoxylin. Slides were digitally scanned using a Nanozoomer (Hamamatsu Nanozoomer 2.0RS, Japan) viewed with the associated NDP view software, with images exported for analysis with Image J^10^. For quantitation, a box (0.4 mm^2^) was placed in four locations within the cortex of each section and all cell bodies greater than 7.5 μm in size, to exclude most non-neuronal cells were counted as either AT180 + ve or –ve. Counts were performed twice and standard deviation between counts was typically < 10%. For double labelling images were incubated NK1 antibody (ab466) and AT180, followed by fluorescent-conjugated secondary antibody (1:250 dilution, Sigma-Aldrich). The slides were counterstained with DAPI for total nuclei counting and imaged with an Olympus BX61 microscope with motorised stage using the imaging software, analySIS Lifescience.

### Statistical methods

Statistical analysis of all data was performed by using GraphPad Prism version 6.0 for Macintosh (GraphPad, La Jolla CA, USA). Normality assumptions were assessed using the D’Agostino and Pearson omnibus normality test. All data was assessed by two-way ANOVA and where overall significance was detected, post hoc Tukey or Dunnett’s t-tests were employed to determine significance at individual treatment levels between timepoints. All data are shown as mean ± SEM and a p value of less than 0.05 was considered statistically significant (Suppl. Fig. S1).

## Supplementary Information

Supplementary Figure S1.

## References

[1] McKee AC, Stein TD, Kiernan PT, Alvarez VE (2015). The neuropathology of chronic traumatic encephalopathy. Brain Pathol..

[2] Feng NH, Lee HH, Shiang JC, Ma MC (2008). Transient receptor potential vanilloid type 1 channels act as mechanoreceptors and cause substance P release and sensory activation in rat kidneys. Am. J. Physiol. Renal Physiol..

[3] Blackshaw LA (2014). Transient receptor potential cation channels in visceral sensory pathways. Br. J. Pharmacol..

[4] Corrigan F, Mander KA, Leonard AV, Vink R (2016). Neurogenic inflammation after traumatic brain injury and its potentiation of classical inflammation. J. Neuroinflamm..

[5] Mantyh PW (2002). Neurobiology of substance P and the NK1 receptor. J. Clin. Psychiatry.

[6] Lai JP (2008). Differences in the length of the carboxyl terminus mediate functional properties of neurokinin-1 receptor. Proc. Natl. Acad. Sci. U S A.

[7] Sun J, Ramnath RD, Tamizhselvi R, Bhatia M (2009). Role of protein kinase C and phosphoinositide 3-kinase-Akt in substance P-induced proinflammatory pathways in mouse macrophages. FASEB J..

[8] Fiebich BL, Schleicher S, Butcher RD, Craig A, Lieb K (2000). The neuropeptide substance P activates p38 mitogen-activated protein kinase resulting in IL-6 expression independently from NF-kappa B. J. Immunol..

[9] Hanger DP, Anderton BH, Noble W (2009). Tau phosphorylation: The therapeutic challenge for neurodegenerative disease. Trends Mol. Med..

[10] Donkin JJ, Nimmo AJ, Cernak I, Blumbergs PC, Vink R (2009). Substance P is associated with the development of brain edema and functional deficits after traumatic brain injury. J. Cereb. Blood Flow Metab..

[11] McAteer KM, Corrigan F, Thornton E, Turner RJ, Vink R (2016). Short and long term behavioral and pathological changes in a novel rodent model of repetitive mild traumatic brain injury. PLoS ONE.

[12] Hoffmann, T., Nimmo, A. J., Sleight, A., Vankan, P., Vink, R. The use of pyridinic NK-1 receptor antagonists for the treatment of brain, spinal or nerve injury. In *Australian Patent WO2003/006016* (2005).

[13] Collins-Praino L, Gutschmidt D, Sharkey J, Arulsamy A, Corrigan F (2018). Temporal changes in tau phosphorylation and related kinase and phosphatases following two models of traumatic brain injury. J. Neuroinflamm. Neurodegener. Dis..

[14] Donkin JJ, Cernak I, Blumberg PC, Vink R (2011). A substance P antagonist reduces axonal injury and improves neurologic outcome when administered up to 12 hours after traumatic brain injury. J. Neurotrauma.

[15] Huber BR (2013). Blast exposure causes early and persistent aberrant phospho- and cleaved-tau expression in a murine model of mild blast-induced traumatic brain injury. J. Alzheimers Dis..

[16] Ang SF, Moochhala SM, MacAry PA, Bhatia M (2011). Hydrogen sulfide and neurogenic inflammation in polymicrobial sepsis: Involvement of substance P and ERK-NF-kappaB signaling. PLoS ONE.

[17] Sun H, Li DP, Chen SR, Hittelman WN, Pan HL (2009). Sensing of blood pressure increase by transient receptor potential vanilloid 1 receptors on baroreceptors. J. Pharmacol. Exp. Ther..

[18] Marchi N (2013). Consequences of repeated blood-brain barrier disruption in football players. PLoS ONE.

[19] Fontaine SN (2015). Cellular factors modulating the mechanism of tau protein aggregation. Cell Mol. Life Sci..

[20] Loane DJ, Kumar A, Stoica BA, Cabatbat R, Faden AI (2014). Progressive neurodegeneration after experimental brain trauma: Association with chronic microglial activation. J. Neuropathol. Exp. Neurol..

[21] Pischiutta F (2018). Single severe traumatic brain injury produces progressive pathology with ongoing contralateral white matter damage one year after injury. Exp. Neurol..

[22] Mouzon BC (2014). Chronic neuropathological and neurobehavioral changes in a repetitive mild traumatic brain injury model. Ann. Neurol..

[23] Lasagna-Reeves CA (2012). Alzheimer brain-derived tau oligomers propagate pathology from endogenous tau. Sci. Rep..

[24] Guerrero-Munoz MJ, Gerson J, Castillo-Carranza DL (2015). Tau oligomers: The toxic player at synapses in Alzheimer's disease. Front. Cell Neurosci..

[25] Cheng JS (2014). Tau reduction diminishes spatial learning and memory deficits after mild repetitive traumatic brain injury in mice. PLoS ONE.

[26] Klomp JE (2016). Mimicking transient activation of protein kinases in living cells. Proc. Natl. Acad. Sci. U S A.

[27] Ahmadzadeh H, Smith DH, Shenoy VB (2014). Viscoelasticity of tau proteins leads to strain rate-dependent breaking of microtubules during axonal stretch injury: predictions from a mathematical model. Biophys. J..

[28] Ma J, Zhang K, Wang Z, Chen G (2016). Progress of research on diffuse axonal injury after traumatic brain injury. Neural Plast..

[29] Sheng JG, Zhu SG, Jones RA, Griffin WS, Mrak RE (2000). Interleukin-1 promotes expression and phosphorylation of neurofilament and tau proteins in vivo. Exp. Neurol..

[30] Vink R (2018). Large animal models of traumatic brain injury. J. Neurosci. Res..

[31] Cernak I (2011). The pathobiology of blast injuries and blast-induced neurotrauma as identified using a new experimental model of injury in mice. Neurobiol. Dis..

[32] Yelverton JT (1996). Pathology scoring system for blast injuries. J. Trauma.

