# Supplementary material for: NK1 antagonists attenuate tau phosphorylation after blast and repeated concussive injury
Source: Sci Rep. 2021 Apr 23;11:8861. doi: 10.1038/s41598-021-88237-0 (PMC8065119; doi:10.1038/s41598-021-88237-0)
Supplement: Supplementary file 1 — Supplementary Figure S1. [file 41598_2021_88237_MOESM1_ESM.pdf]

NK1 antagonists attenuate tau phosphorylation after blast and repeated concussive injury

Frances Corrigan[1,2], Ibolja Cernak[3], Kelly McAteer[2], Sarah C. Hellewell[3], Jeffrey V. Rosenfeld[4,5], Renée J. Turner[2] and Robert Vink[1]

[1]School of Health Sciences, University of South Australia, Adelaide, Australia; [2]Discipline of Anatomy and Pathology, Adelaide Medical School, University of Adelaide, Adelaide, Australia; [3]Department of Biomedical Sciences, Mercer University School of Medicine, Macon, Georgia, USA; [4]Department of Surgery, Monash University, Melbourne, Australia; [5]Department of Neurosurgery, The Alfred Hospital, Melbourne, Australia.

Supplementary material. Complete Western blots for phospho-tau (serine 396), total tau (tau5) and pyruvate kinase loading control for sham, 24 h and the 28-day timepoint mice as presented in manuscript Figure 3.

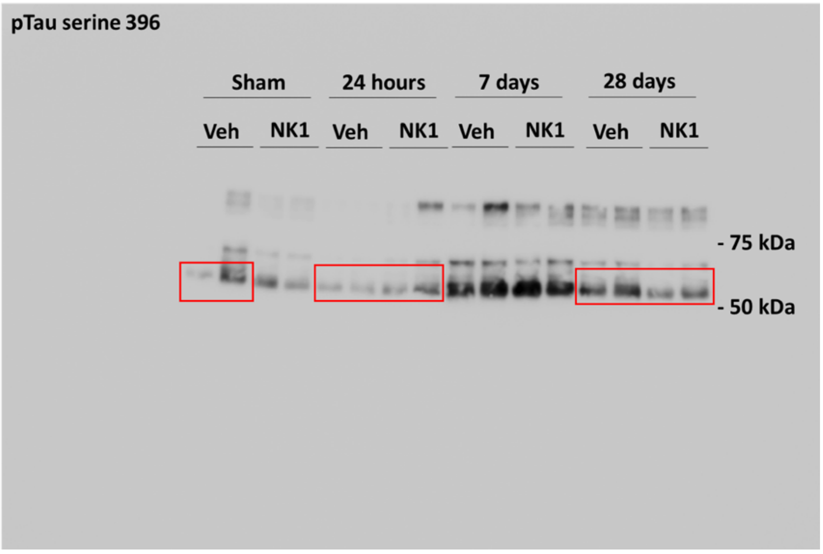

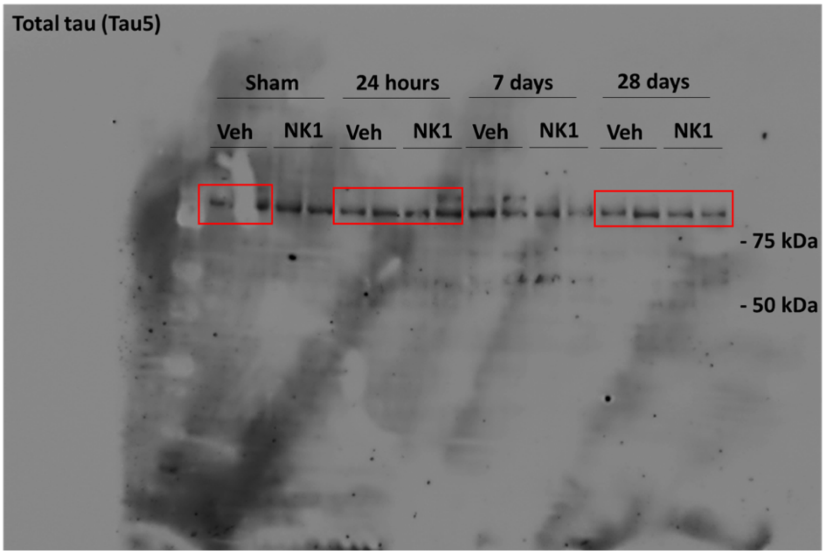

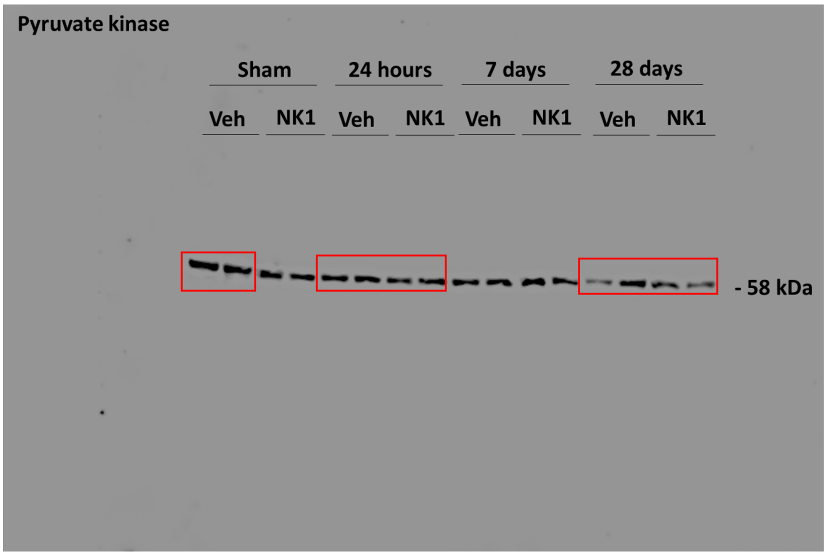

Supplementary Figure 1. Complete Western blots for phospho-tau (serine 396), total tau (tau5) and pyruvate kinase loading control for sham, 24 h and the 28-day timepoint mice as presented in manuscript Figure 3. Corresponding regions of the blot in figure 3A are highlighted with red boxes, which have also been provided to aid identification of corresponding animals on Tau5 and pyruvate kinase blots. Samples included in the manuscript were run alongside samples from a 7-day timepoint, which was not presented in this current manuscript. Veh = vehicle treatment, NK1 = treatment with the NK1 antagonist EUC-001.
